# Supplementary material for: Assembly and annotation of an Ashkenazi human reference genome
Source: Genome Biol. 2020 Jun 2;21:129. doi: 10.1186/s13059-020-02047-7 (PMC7265644; doi:10.1186/s13059-020-02047-7)
Supplement: Supplementary file 2 — Additional file 2:Figure S1, S2, and S3. [file 13059_2020_2047_MOESM2_ESM.docx]

**Supplementary Figures**

GRCh38 (yellow)

Ash1 (green) (b) (c) (a)

100bp gaps

**Figure S1**. Adding sequences from GRCh38 to the Ash1 genome assembly. In (a), GRCh38 closes a gap in Ash1. In (b), the GRCh38 contig extends into a gap in Ash1, but the sequence adjacent to the gap does not match. If the GRCh38 extended >1000bp into the gap, and if the alignment ended > 100bp from the end of the Ash1 contig, then the GRCh38 sequence indicated by (b) was inserted, separated from the Ash1 sequence by a gap set to 100 Ns. Case (c) shows an example where a separate GRCh38 contig falls completely within a gap in Ash1, in which case it would be inserted with gaps on both sides.

**
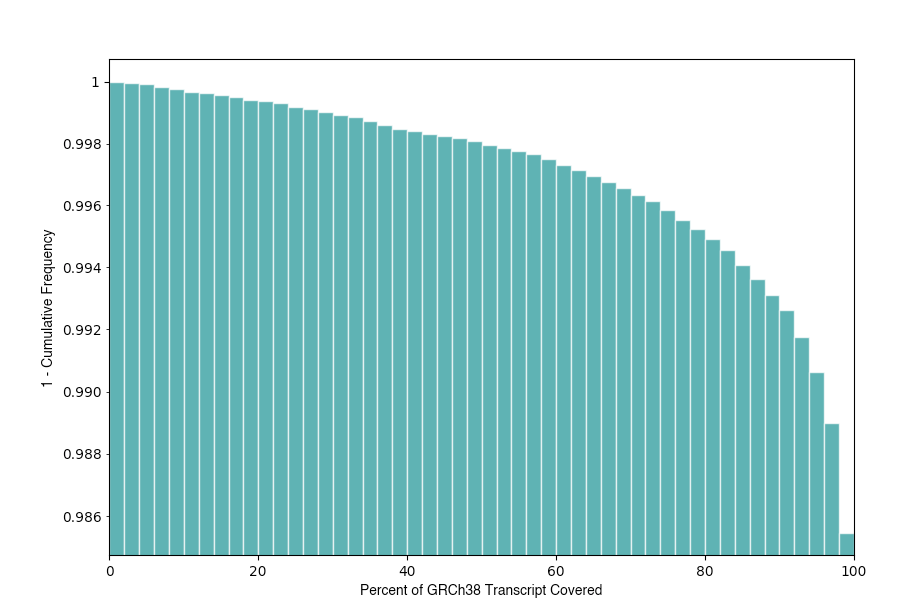
**

**Figure S2**. Cumulative distribution showing how much of the GRCh38 transcripts map onto Ash1. The Y axis shows the fraction of transcripts with percent coverage greater than or equal to coverage on the X axis; e.g., the next-to-last bar at 98% on the X axis shows that 98.9% of GRCh38 transcripts (Y axis) mapped for at least 98% of their length onto Ash1.

**
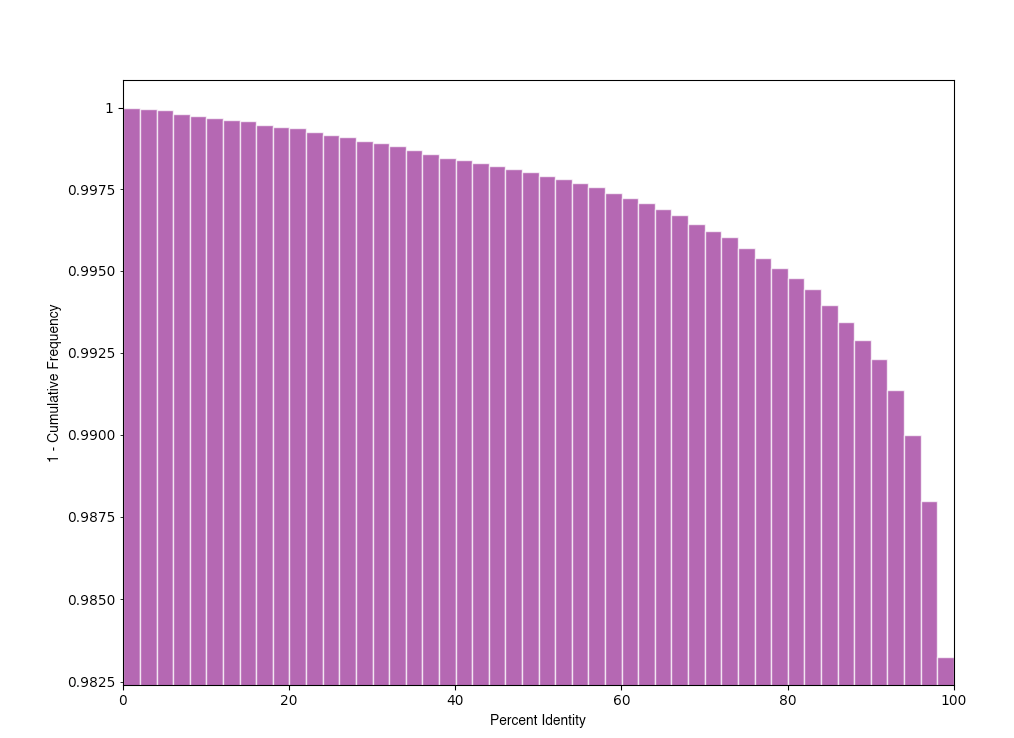
**

**Figure S3.** Cumulative distribution of the sequence identity of transcripts mapped onto Ash1. The Y axis shows the fraction of transcripts that aligned between GRCh38 and Ash1 with DNA sequence identity greater than or equal to the percent identity on the X axis. E.g., the next-to-last vertical bar at 98% on the X axis shows that 98.75% of the GRCh38 transcripts aligned at 98% or greater identity to Ash1.
