# Supplementary material for: Assembly and annotation of an Ashkenazi human reference genome
Source: Genome Biol. 2020 Jun 2;21:129. doi: 10.1186/s13059-020-02047-7 (PMC7265644; doi:10.1186/s13059-020-02047-7)
Supplement: Supplementary file 3 — Additional file 3. Review history. [file 13059_2020_2047_MOESM3_ESM.docx]

Review History

**First round of review**

**Reviewer 1**

**Are you able to assess all statistics in the manuscript, including the appropriateness of statistical tests used?**

There are no statistics in the manuscript.

**Comments to author:**

Shumate and colleagues provide a well-written manuscript presenting an accurate assembly of a new human reference genome derived from an individual of Ashkenazi ancestry. The argument for alternative human reference genomes is quite clear given the extreme genetic diversity that exists in the human population but is not well capture in the haploid GRCh38 genome that reflects a consensus sequence ascertained from a handful of humans. Overall, the authors do a nice job demonstrating the quality of the assembly and provide nice methods that can be used as a template for new reference genomes from other humans.

My main constructive criticism is that the manuscript does not make a terribly strong case for the utility of this new genome for variant discovery and disease over GRCh38. In particular, the section entitled "Comparison of variant calling using Ash1 versus GRCh38" focuses solely on sites where the Ashkenazi sample PGP17 is homozygous. Not surprisingly, fewer sites were homozygous differences with Ash1 than GRCh38 given the shared ancestry. The authors find that of the 1.33 million such differences between PGP17 and Ash1, "approximately half of them" Ash1 was actually heterozygous for the same allele, yet the consensus reference sequence belied that heterozygous truth in Ash1. I struggle to be compelled by this analysis, as a researcher is going to be more interested in two questions that do not seem to be addressed in the manuscript. First, what _new_ variants are found in PGP17 by alignment to Ash1 than GRCh38. Second, how many PGP17 genotypes are correctly reported in Ash1 that were misreported when compared to GRCh38? These latter two questions are the critical motivations for using this reference to study new genomes (of Ashkenazi ancestry) in disease research. I worry that the analysis they conducted is relatively easily addressed when aligning to GRCh38 and comparing variants to what is variants/alleles in gnomAD, which includes individuals of Ashkenazi ancestry. If I am missing an essential point, I suggest that the authors strive to make the rationale for choosing Ash1 over GRCh38 clearer.

Minor points:

1. "Because the genetic background is similar, fewer variants should be found when searching against Ash1, and the variants that do not appear will be more likely to be disease-relevant". I agree with the first clause of this statement, but I do not follow the logic behind the second clause at all. Why should these variants be more likely to be disease relevant?

2. Change "slightly more reads (3,901,270, 0.5%) to "A slightly higher fraction of reads"

**Reviewer 2**

**Are you able to assess all statistics in the manuscript, including the appropriateness of statistical tests used?**

Yes, and I have assessed the statistics in my report.

**Comments to author:**

Dear authors,

I enjoyed reading the manuscript from Shumate and Zimin et al. In this work, the authors have created Ash1, an HG002-based reference genome. The methods for assembly and annotation have been well executed and validated using GIAB and CHESS datasets. I also appreciate that the authors preserved the conventional coordinate systems for annotation which will further increase the reach of their work. This work will serve as a reference genome to the community.

I have a couple of minor comments that are mostly curiosity:

1. Did the authors use the long read nanopore data for HG002 from Genome in a Bottle (https://github.com/genome-in-a-bottle/giab_data_indexes/blob/master/AshkenazimTrio/sequence.index.AJtrio_UCSC_ONT_UL_Promethion_03312019.HG002)?

2. It may also be useful to include the access information for the raw sequence data across all platforms from GIAB?

3. Would the scaffolding benefit from HiC data vs. using GRCh38?

4. Long-term, how would phasing play into this?

My best wishes to the authors.

**Authors Response**

**Point-by-point responses to the reviewers’ comments:**

Reviewer reports:

Reviewer #1: […]

My main constructive criticism is that the manuscript does not make a terribly strong case for the utility of this new genome for variant discovery and disease over GRCh38. In particular, the section entitled "Comparison of variant calling using Ash1 versus GRCh38" focuses solely on sites where the Ashkenazi sample PGP17 is homozygous. Not surprisingly, fewer sites were homozygous differences with Ash1 than GRCh38 given the shared ancestry. The authors find that of the 1.33 million such differences between PGP17 and Ash1, "approximately half of them" Ash1 was actually heterozygous for the same allele, yet the consensus reference sequence belied that heterozygous truth in Ash1. I struggle to be compelled by this analysis, as a researcher is going to be more interested in two questions that do not seem to be addressed in the manuscript. First, what _new_ variants are found in PGP17 by alignment to Ash1 than GRCh38. Second, how many PGP17 genotypes are correctly reported in Ash1 that were misreported when compared to GRCh38? These latter two questions are the critical motivations for using this reference to study new genomes (of Ashkenazi ancestry) in disease research. I worry that the analysis they conducted is relatively easily addressed when aligning to GRCh38 and comparing variants to what is variants/alleles in gnomAD, which includes individuals of Ashkenazi ancestry. If I am missing an essential point, I suggest that the authors strive to make the rationale for choosing Ash1 over GRCh38 clearer.

*Thanks for these constructive comments. In response, we first want to emphasize that the main benefit of a population-specific genome is not that one finds new variants, but rather than one finds many fewer variants when scanning a new genome (such as PGP17) for SNPs or other variants. This is because common differences between the background population (here, Ashkenazi Jews) and GRCh38 simply won’t show up when you align PGP17 to Ash1. In genetic studies, one of the main challenges is sorting through millions of variants to find the very small number (often just a handful) that are correlated with the disease or other trait of interest. Thus if your analysis begins with fewer variants, it should be simplified because you don’t have to rule out as many common population variants.*

*The reviewer makes a good point, though, in arguing that one could align to GRCh38 first and then remove variants/alleles by using gnomAD. This two-step process would be more complex, but it might yield similar results. It would fall short for regions of Ash1 that are simply missing from GRCh38 (a relatively small amount, but still >30 Mbp)..*

*As the reviewer suggests, we may not have made these points clear enough, so we’ve added text (but not too much) to the section in question to clarify that the main benefit is in finding fewer variants. At the end of the section (p. 6, end of first paragraph), we updated the text to say this:*

*Original: “Thus we found just under 1 million fewer homozygous SNVs when using Ash1 as the reference for PGP17.”*

*Revised: “Thus we found just under 1 million fewer homozygous SNVs when using Ash1 as the reference for PGP17. Note that rather than aligning to Ash1, one could instead align the reads to GRCh38 and then remove known population-specific variants. This two-step process, although more complex, might yield similar results, except for regions of Ash1 that are simply missing from GRCh38.”*

*The reviewer also asked how many new variants we found in PGP17 by aligning to Ash1. This is surprisingly hard to answer: we couldn’t simply compare the VCF files, because the coordinate systems are different. We’d have to go back and compare the original alignments for all reads supporting each SNV. However, a variant in PGP17 found by alignment to Ash1 but not GRCh38 may simply represent a place in PGP17 that is non-Ashkenazi, which covers 33% of PGP17. So we don’t want to argue that finding such a variant is an advantage here–indeed, most such variants are probably common population variants as well, just from a different population. Thus we have chosen not to highlight any such new variants in our manuscript, because they aren’t part of our argument for the benefits of Ash1. We hope this is clearer now.*

*To add one more note of emphasis, in the following section we made another small revision. That section describes how we matched the major Ashkenazi alleles from gnomAD to Ash1, and then replaced many of them as long as the Ash1 read data supported the major allele. We didn’t explicitly say that this step also reduces the number of variants one might find when aligning an Ashkenazi individual, so we made note of that. The addition appears at top of p. 7, at the end of the paragraph explaining these replacements with major alleles, where we added:*

*“These modifications should further reduce the number of reported SNVs when aligning an Ashkenazi individual to Ash1.”*

Minor points:

1. "Because the genetic background is similar, fewer variants should be found when searching against Ash1, and the variants that do not appear will be more likely to be disease-relevant". I agree with the first clause of this statement, but I do not follow the logic behind the second clause at all. Why should these variants be more likely to be disease relevant?

*Response: rather than trying to elaborate, which might require a lengthy digression, we simply deleted the second clause.*

2. Change "slightly more reads (3,901,270, 0.5%) to "A slightly higher fraction of reads"

*Done.*

Reviewer #2: […] I have a couple of minor comments that are mostly curiosity:

1. Did the authors use the long read nanopore data for HG002 from Genome in a Bottle (https://github.com/genome-in-a-bottle/giab_data_indexes/blob/master/AshkenazimTrio/sequence.index.AJtrio_UCSC_ONT_UL_Promethion_03312019.HG002)?

*Response: No, this long-read is a very recent data set, which we did not use for assembly. However, we did use them for the independent validation illustrated in Figure 1, and we provide a link to the data source in the Methods section. We are working on an improved assembly that will incorporate this data, for future releases.*

2. It may also be useful to include the access information for the raw sequence data across all platforms from GIAB?

*We already provide several references to GIAB, but because GIAB can move the raw data, we instead provide the NCBI ftp site information. Our understanding is that NCBI is working directly with GIAB to provide stable URLs.*

3. Would the scaffolding benefit from HiC data vs. using GRCh38?

*The short answer is that scaffolding might benefit from HiC data, if that data came from HG002. If it came from GRCh38, it might lead to incorrect scaffolds where there are structural differences between HG002 and GRCh38. We already have chromosome-length scaffolds, though, so the primary places that need improvement are the very difficult-to-assemble regions including centromeres and a few other locations. We plan to continue improving Ash1 in the future using whatever technology allows us to finish those regions.*

4. Long-term, how would phasing play into this?

*For now, we chose to produce a single sequence for each chromosome, just as GRCh38 is represented. Many groups are working on diploid genome assemblies, where both haplotypes are assembled for each of the autosomes. If that can be done correctly, then (in the long-term, as the reviewer asks) we might produce a true diploid version of Ash1, with two copies of each chromosome. Because almost all analysis tools today operate on a haploid genome, such an assembly wouldn’t yet be useful for most applications. We could also replace the chromosomes with just one copy that represented a single haplotype, if we had an accurately phased assembly. We instead chose to use the major allele (from gnomAD) for the population in places where Ash1 was heterozygous. We think this will prove better, for now, for short-read mapping to Ash1.*
